# Individualized positive end-expiratory pressure guided by driving pressure in robot-assisted laparoscopic radical prostatectomy: a prospective, randomized controlled clinical trial

**DOI:** 10.3389/fmed.2025.1573150

**Published:** 2025-04-22

**Authors:** Yanfang Luo, Siyu Qin, Mengxiao Liu, Qian Shen, Ran An, Yan Jiang

**Affiliations:** Department of Anesthesiology, Chongqing University Cancer Hospital, Chongqing, China

**Keywords:** positive end-expiratory pressure, driving pressure, pulmonary gas exchange, lung protection ventilation, intracranial hypertension, postoperative delirium, radical prostatectomy

## Abstract

**Background:**

Despite the widespread use of lung-protective ventilation in general anesthesia, the optimal positive end-expiratory pressure (PEEP) remains uncertain. This study aimed to investigate the effects of driving pressure-guided individualized PEEP in patients undergoing robot-assisted laparoscopic radical prostatectomy.

**Methods:**

Forty-two male patients undergoing robot-assisted laparoscopic radical prostatectomy were randomized to receive conventional fixed PEEP of 5 cmH_2_O (*n* = 21, PEEP_5_) or driving pressure-guided individualized PEEP (*n* = 21, PEEP_IND_). The primary outcome was the ratio of arterial oxygen partial pressure to fractional inspired oxygen (PaO_2_/FiO_2_). The secondary outcomes included respiratory mechanics, hemodynamics, optic nerve sheath diameter (ONSD), and the incidence of postoperative delirium (POD) and postoperative pulmonary complications (PPCs) within a 7-day period.

**Results:**

In comparison with the PEEP_5_ group, the PEEP_IND_ group showed significantly higher (*p* < 0.001) PEEP values during pneumoperitoneum in the Trendelenburg position (mean [standard deviation], 11.29 cmH_2_O [1.01 cmH_2_O]) and after deflation and repositioning to the supine position (mean [standard deviation], 7.05 cmH_2_O [1.20 cmH_2_O]). The PaO_2_/FiO_2_ values in the PEEP_IND_ group were significantly higher than those in the PEEP_5_ group 120 min after pneumoperitoneum in the Trendelenburg position (*p* = 0.023) and at the end of the operation (*p* = 0.028). The groups showed no differences in ONSD, hemodynamics, and incidence of POD and PPCs (*p* > 0.05).

**Conclusion:**

In comparison with a fixed PEEP of 5 cmH_2_O, driving pressure-guided individualized PEEP improves intraoperative respiratory mechanics and oxygenation without causing deterioration in hemodynamics, further escalation in intracranial pressure, or an increase in the incidence of POD. Nevertheless, this procedure requires meticulous monitoring. Unfortunately, individualized PEEP did not result in a reduction in the incidence of PPCs in this study.

**Clinical Trial Registration:**

http://www.chictr.org.cn, ChiCTR2400081338.

## 1 Introduction

Robot-assisted laparoscopic radical prostatectomy has become an increasingly popular procedure among surgeons due to several advantages, including minimal surgical trauma and blood loss, preservation of nerve structures, and facilitation of faster postoperative recovery (1). However, it is important to note that pneumoperitoneum and the steep Trendelenburg position, which are essential for achieving adequate surgical exposure, can elevate intra-abdominal pressure. This, in turn, can exacerbate atelectasis during general anesthesia, leading to detrimental effects on respiratory mechanics (2). Positive end-expiratory pressure (PEEP) plays a critical role in preventing small-airway collapse, maintaining alveolar patency, reducing atelectasis, and improving lung function (3). However, conventional fixed PEEP of 5 cmH₂O is often insufficient to prevent alveolar collapse under conditions of elevated intra-abdominal pressure and diaphragmatic displacement (4, 5), necessitating higher PEEP levels in certain scenarios. Studies suggested that PEEP up to 15 cmH₂O may be required to mitigate airway collapse and ventilation heterogeneity induced by the Trendelenburg position (6, 7). However, excessive PEEP carries risks of lung hyperinflation, inflammatory mediator release, and hemodynamic compromise (7, 8). Given the significant inter-individual variability in optimal PEEP levels, a standardized “one-size-fits-all” approach is suboptimal (9). Therefore, individualized PEEP (PEEP_IND_), tailored to patient-specific factors, pneumoperitoneum pressure, and body position, may provide superior lung protection compared to fixed PEEP strategies.

Although various methods can be used for titrating individualized PEEP (4, 9–12), driving pressure-guided individualized PEEP titration can be performed without any special equipment other than the anesthesia machine. Studies have demonstrated that the incidence of postoperative pulmonary complications (PPCs) can be reduced exclusively by modifying ventilatory parameters with the objective of reducing driving pressure (13, 14). Consequently, a ventilation strategy involving individualized titration of PEEP guided by a minimum driving pressure has the potential to optimize intraoperative respiratory mechanics, improve oxygenation, reduce PPCs, and promote recovery. Kim et al. (15) found that the driving pressure-guided PEEP group showed improved intraoperative oxygenation, but did not show a reduction in the incidence of PPCs. However, in clinical practice, we found that in decremental PEEP trials guided by driving pressure, the minimum driving pressure corresponded to a range of PEEP values rather than a specific point, which had not been explicitly stated in previous studies. Therefore, the present study defined the minimum PEEP value within the specified range corresponding to the lowest driving pressure as the individualized PEEP (PEEP_IND_) and investigated whether it could improve oxygenation and postoperative recovery.

Pneumoperitoneum and the Trendelenburg position have been observed to cause a series of physiological changes in patients, including alterations to the respiratory and cardiovascular systems as well as an increased intracranial pressure (ICP) (16, 17). An elevated ICP could result in delayed emergence from general anesthesia, postoperative delirium (POD), and a decline in cognitive function (18–20). Non-invasive ocular sonography is a well-established method of evaluating ICP (21–23). The issue of whether supplying patients with individualized PEEP will lead to further disruption of hemodynamics and the exacerbation of the rise in intracranial pressure, potentially leading to POD, remains to be evaluated.

## 2 Patients and methods

This single center randomized controlled trial was approved by the ethics committee of Chongqing University Cancer Hospital and registered at http://www.chictr.org.cn (registration No.: ChiCTR2400081338) on 28/02/2024. This study was conducted from March 2024 to November 2024, and informed consent was obtained from all patients before enrolment.

### 2.1 Participants

#### 2.1.1 Inclusion criteria

The study population consisted of patients scheduled to undergo robot-assisted laparoscopic radical prostatectomy who were aged ≥18 years and had BMI < 30 and >18.5 kg/m^2^, American Society of Anesthesiologists physical status I-III, and a moderate or high risk of PPCs based on a Assess Respiratory Risk in Surgical Patients in Catalonia (ARISCAT) risk score ≥ 26.

#### 2.1.2 Exclusion criteria

Patients were excluded from the study if they had experienced heart failure (New York Heart Association classification III or greater), had a history of severe cardiopulmonary diseases, atrial fibrillation, neuromuscular dysfunction, increased ICP or glaucoma, preoperative mini-mental state examination score < 24, undergone conversion to open approach, or showed life-threatening complications due to intraoperative hemorrhage.

#### 2.1.3 Randomization and blinding

**Figure 1:**
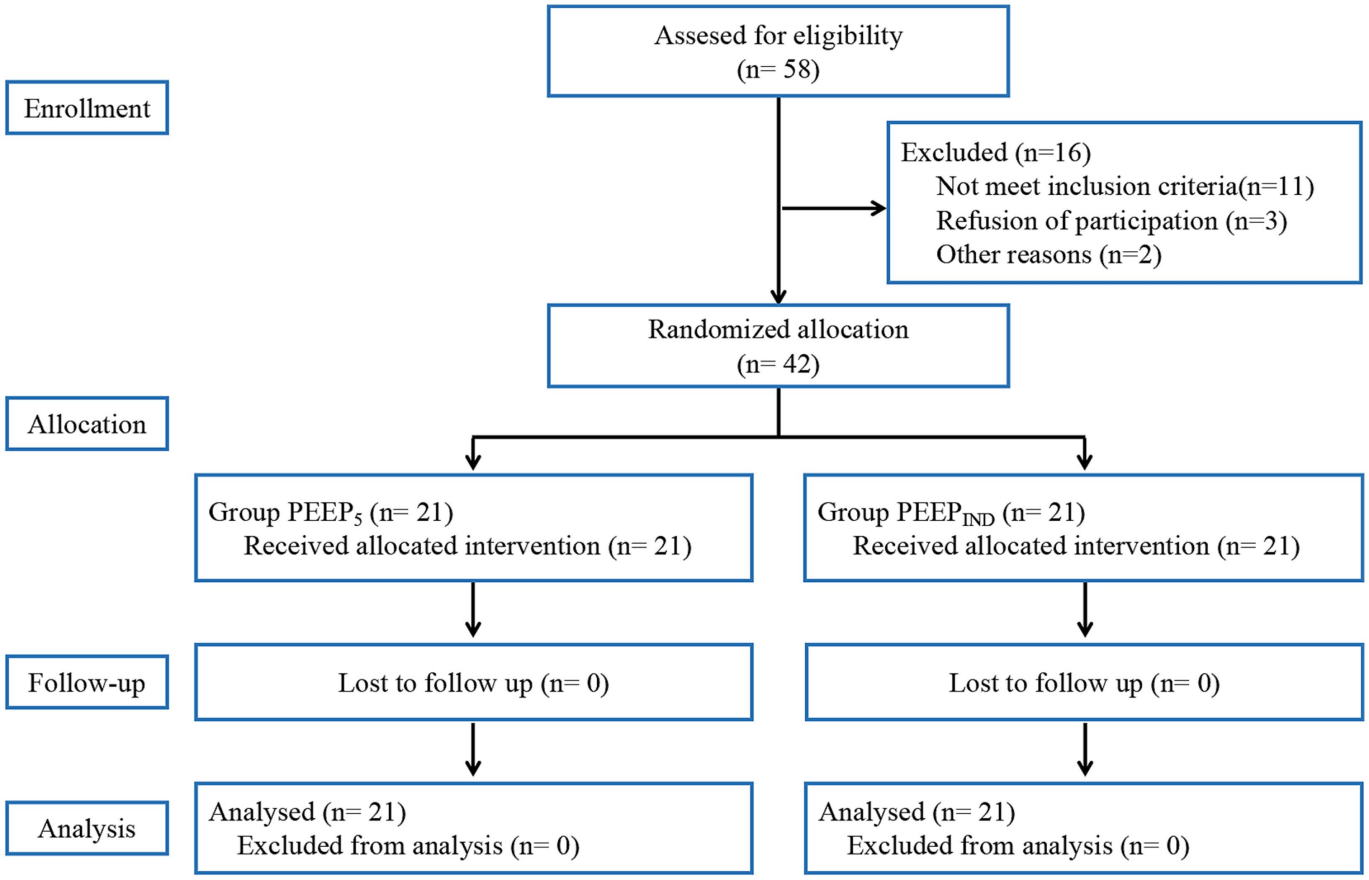
Flow chart of the study. PEEP_5_, positive end-expiratory pressure of 5 cmH_2_O; PEEP_IND_, individualized positive end-expiratory pressure.

The randomization sequence was generated using a computer program by an investigator not involved in the study. A total of 58 patients who underwent robot-assisted laparoscopic radical prostatectomy were recruited, and 42 patients were eventually enrolled and randomized into two groups: a conventional group that received fixed PEEP of 5 cm H_2_O (PEEP_5_ group, *n* = 21) and an individualized PEEP group in which PEEP was guided by the minimum driving pressure (PEEP_IND_ group, *n* = 21). The random allocation sequence was sealed in an opaque envelope and released to the attending anaesthesiologist immediately before the trial. The surgeons, patients, and independent investigators who performed the data collection and analysis were all blinded to group allocation, but the attending anaesthesiologist was not blinded to study group allocation. The flow chart of the study is shown in Figure 1.

### 2.2 Study protocol

After admission to the operating theater, the patient underwent routine monitoring of peripheral pulse oximetry, electrocardiography, non-invasive blood pressure, and heart rate data, and a peripheral venous catheter was inserted to allow fluid infusion. In addition, an arterial cannula was inserted and connected to Most-care (Vytech health, PROJECT ENGINEERING, Italy) for continuous monitoring of blood pressure, cardiac index, and stroke volume variation and for arterial blood gas sampling.

After induction of anesthesia and endotracheal intubation, patients were mechanically ventilated (WATO EX—65 Pro, Mindray, China) in a volume-controlled mode with PEEP of 5 cmH_2_O. Other settings included a tidal volume of 6–8 mL/kg of predicted body weight (50 + 0.91 × [height (cm) – 152.4]), 20% inspiratory pause, inspiratory oxygen fraction of 0.4, inspiratory to expiratory (I: E) ratio of 1:2, and a respiration rate adjusted for an end-tidal carbon dioxide partial pressure in the range of 35–45 mmHg. Patients received volume expansion to maintain the stroke volume variation (SVV) to less than 13% prior to the recruitment maneuver (RM). In both groups, the first RM was performed after intubation. After the first RM, the PEEP was decreased to 5 cmH₂O in the PEEP_5_ group and then maintained throughout the surgery. In the PEEP_IND_ group, a decremental PEEP-titration trial (volume-controlled ventilation mode as mentioned above) was then initiated immediately at the end of the first RM. During this trial, the minimum driving pressure was documented by reducing PEEP in 2-cmH₂O decrements from 20 cmH₂O to 4 cmH₂O. The minimal PEEP corresponding to the minimum driving pressure was defined as the PEEP_IND_. Following PEEP titration, a second RM was performed immediately in the PEEP_IND_ group. Subsequent mechanical ventilation was then conducted with PEEP_IND__1 (defined as PEEP_IND_ level determined in supine position).

**Figure 2:**
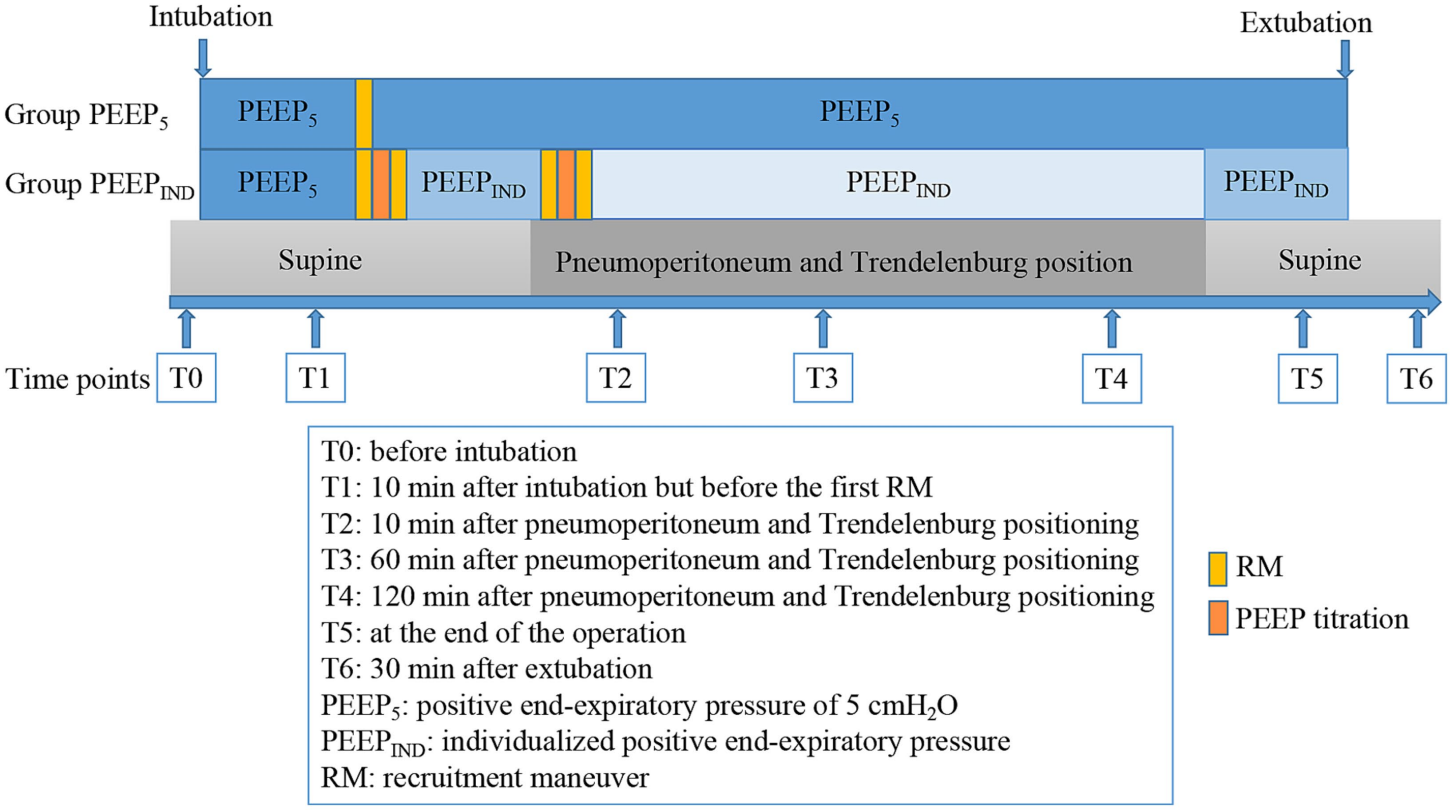
Timeline of the study protocol and interventions for both groups.

For patients in the PEEP_IND_ group, the third RM was initiated after establishing a pneumoperitoneum of 12 mmHg in the 25° Trendelenburg position. At the end of the RM, an additional PEEP-titration trial for PEEP_IND__2 was initiated, which was immediately followed by a fourth RM. The patient was then mechanically ventilated with PEEP_IND__2 until the pneumoperitoneum was deflated and the patient was repositioned in the supine position. PEEP_IND__2 was then adjusted to PEEP_IND__1 for mechanical ventilation until the end to prevent further lung hyperinflation. The tidal volume was maintained unchanged until extubation in both groups. All RMs were uniformly performed in pressure-controlled ventilation mode, with a gradient of 20 cmH₂O in airway pressure and PEEP. Specifically, PEEP was initiated at 5 cmH₂O and gradually increased by 5 cmH₂O every three respiratory cycles, up to a maximum of 20 cmH₂O. Simultaneously, the airway pressure was increased stepwise to 30, 35, and 40 cmH₂O, and maintained at 40 cmH₂O for six respiratory cycles. The entire recruitment process was completed within 90 s (7). The study protocol timeline and interventions are shown in Figure 2.

Total intravenous anesthesia was adopted in this study. Propofol (2–3 μg/mL) and remifentanil (2–4 ng/mL) in target-controlled infusion mode were applied for the maintenance of general anesthesia; sufentanil 5–10 μg was administered when necessary; and muscle relaxants were administered in a stepwise fashion under the guidance of a neuromuscular monitor. The bispectral index was maintained at 40–55, and the nasopharyngeal temperature was maintained at 36°C to 37°C during anesthesia. In the event of hypotension (systolic blood pressure < 90 mmHg) or bradycardia (HR < 50 bpm), vasoactive drugs were administered intravenously as appropriate. Intraoperative data were collected, including variables such as duration of surgery and anesthesia, duration of pneumoperitoneum and Trendelenburg position, volume of fluid infusion, bleeding and urine output, and dosage of vasoactive drugs.

### 2.3 Outcome measures

The following time points were designated for collection of data: before intubation (T0); 10 min after intubation but before the first RM (T1); 10 min after pneumoperitoneum and Trendelenburg positioning (T2); 60 min after pneumoperitoneum and Trendelenburg positioning (T3); 120 min after pneumoperitoneum and Trendelenburg positioning (T4); at the end of the operation (T5); and 30 min after extubation (T6). The time points for data collection are shown in Figure 2.

#### 2.3.1 Primary outcome

The primary outcome was the PaO_2_/FiO_2_ ratio. Arterial blood gas samples for analysis of PaO_2_/FiO_2_ were collected at T0, T1, T4, T5, and T6.

#### 2.3.2 Secondary outcomes

Data pertaining to pulmonary variables (airway peak pressure, airway plateau pressure, respiratory system compliance, driving pressure, PEEP) and hemodynamic data (mean arterial pressure, cardiac index, and stroke volume variation) were collected at T1, T2, T3, T4, and T5. The driving pressure is defined as the tidal volume divided by the respiratory system compliance and can be readily calculated as the plateau pressure minus the PEEP (24). Arterial blood gas samples for analysis of arterial partial pressure of carbon dioxide (PaCO_2_) were collected at T0, T1, T4, T5, and T6.

Optic nerve sheath diameter (ONSD) was measured by an investigator trained in ocular sonography who was blinded to the group assignment. ONSD was measured 3 mm behind the sphere at three time points (T1, T4, and T5) using a high-frequency linear-array ultrasound probe (UMT −500; Mindray, China). This process was repeated for both eyes, with three measurements taken for each. The average value was obtained as the patient’s ONSD.

The occurrence of complications within 7 days after surgery was meticulously documented. These complications were defined as follows: hypoxemia (SpO₂ < 90%), the necessity for postoperative oxygen therapy on day 2 or later, initial ventilatory support for a period exceeding 24 h, re-intubation and mechanical ventilation, pneumonia, pneumothorax, pulmonary failure, and POD. The Confusion Assessment Method was used to assess POD twice a day with a 6-h interval from day 1 to 7 after surgery.

### 2.4 Statistical analysis

The sample-size calculation was based on detecting differences of 100 mmHg in PaO_2_/FiO_2_ between the two ventilation strategies, with an SD of 90 mm Hg in each arm (4). A total sample size of 38 participants was needed to achieve a study power of 90% with a 5% alpha error. Allowing for a 10% rate of incomplete follow-up or dropout, at least 42 patients were required in this study.

Statistical analysis was performed using SPSS software (version 25.0, IBM Corp, Armonk, NY). The Shapiro–Wilk test was used to assess the normality of the distributions. Continuous data were analyzed using the independent Student *t* test or the Manne-Whitney U test. Pearson’s χ^2^ test or Fisher’s exact test was used to compare categorical data where appropriate. Two-way analysis of variance (ANOVA) followed by the Bonferroni *post-hoc* test was used for repeated-measures data. A value of *p* < 0.05 was considered statistically significant.

## 3 Results

### 3.1 Demographic and clinical characteristics

The two groups showed no significant differences in demographic and clinical characteristics (Table 1).

**Table 1:** The demographic and clinical characteristics of patients.

Baseline characteristics	Group PEEP_5_	Group PEEP_IND_	*P* value
Age (y)	67.00 ± 6.88	66.48 ± 6.21	0.797
Predicted body weight (kg)	66.64 ± 6.98	67.81 ± 8.04	0.618
Body mass index (kg/m^2^)	24.97 ± 2.48	25.18 ± 3.10	0.805
ASA physical status			0.525
II	9 (42.9)	7 (33.3)	
III	12 (57.1)	14 (66.7)	
ARISCAT score	26 (8)	26 (4)	0.636
**Pre-existing medical condition**			
Smoking			0.931
Never	7 (33.3)	6 (28.6)	
Former	7 (33.3)	8 (38.1)	
Current	7 (33.3)	7 (33.3)	
Hypertension	7 (33.3)	11 (52.4)	0.212
Diabetes	3 (14.3)	5 (23.8)	0.697
Coronary heart disease	3 (14.3)	4 (19.0)	1.000
Sleep apnea	2 (9.9)	2 (9.9)	1.000
**Intraoperative characteristic**			
Anesthesia duration (min)	311.10 ± 79.74	311.24 ± 80.29	0.995
Surgery duration (min)	265.86 ± 79.63	260.81 ± 65.56	0.824
Pneumoperitoneum duration (min)	223.29 ± 67.38	218.81 ± 67.92	0.831
Infusion volume (mL)	2665.48 ± 716.30	2498.81 ± 676.13	0.443
Bleeding (mL)	97.62 ± 66.09	88.57 ± 44.64	0.606
intraoperative output (mL)	714.29 ± 266.52	764.29 ± 232.99	0.521
Ephedrine (mg)	7.19 ± 5.98	6.33 ± 4.95	0.616
Norepinephrine (μg)	720.78 ± 450.86	602.17 ± 283.82	0.314

Data are presented as mean ± standard deviation, median (inter-quartile range), or n (%). PEEP_5_, positive end-expiratory pressure of 5 cmH_2_O; PEEP_IND_, individualized positive end-expiratory pressure; ARISCAT, Assess Respiratory Risk in Surgical Patients in Catalonia; ASA, American Society of Anesthesiologists.

### 3.2 Primary outcome—PaO_2_/FiO_2_

**Figure 3:**
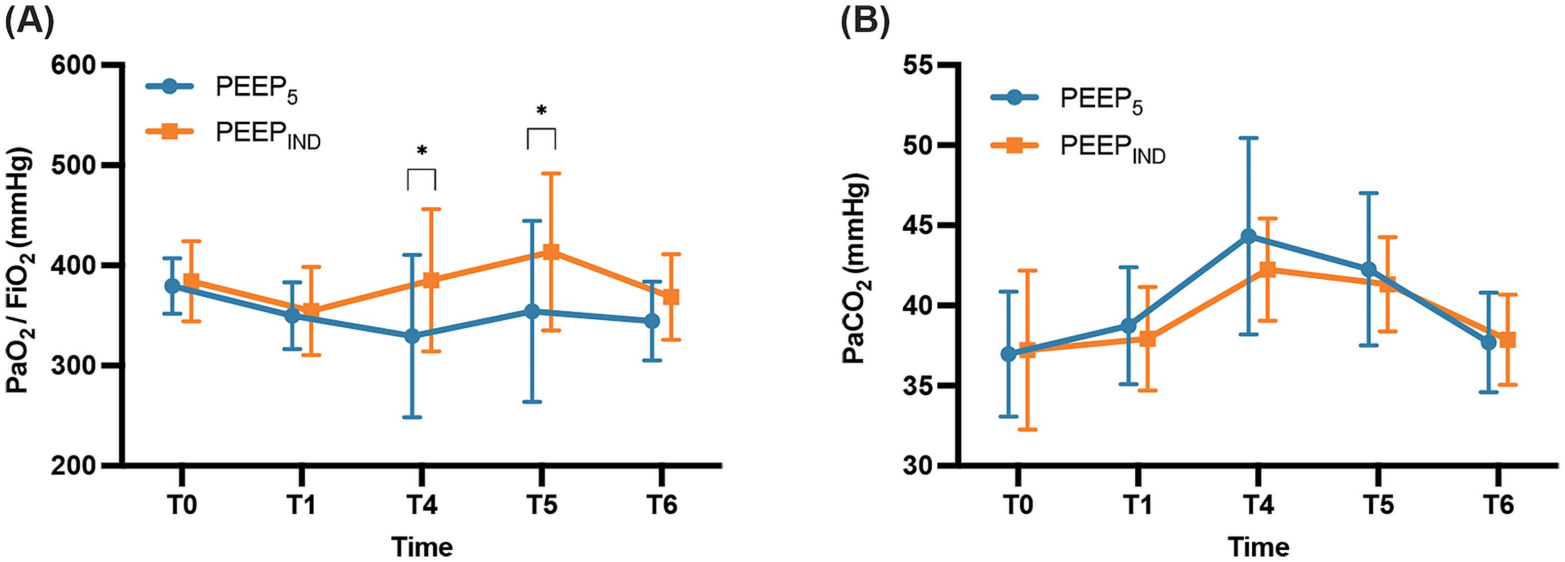
Arterial blood gas parameters at five time points. **(A)** PaO_2_/FiO_2_ ratio. **(B)** PaCO_2_. T0, before intubation; T1, 10 min after intubation but before the first RM; T4, 120 min after pneumoperitoneum and Trendelenburg positioning; T5, at the end of the operation; T6, 30 min after extubation. ^*^p < 0.05.

A two-way repeated-measures ANOVA was performed to evaluate the PaO_2_/FiO_2_ at five designated time points. The findings demonstrated no difference in PaO_2_/FiO_2_ between the two groups neither before nor after intubation (*p* > 0.05). PaO_2_/FiO_2_ was significantly higher 120 min after pneumoperitoneum in the Trendelenburg position (*p* = 0.023) and at the end of the operation (*p* = 0.028) in the PEEP_IND_ group than in the PEEP_5_ group. However, no intergroup difference was found in the PaO_2_/FiO_2_ 30 min after extubation (*p* > 0.05) (Figure 3). The PaCO_2_ showed no significant difference between the two groups (*p* > 0.05) (Figure 3).

### 3.3 Secondary outcomes

#### 3.3.1 Respiratory mechanics

**Figure 4:**
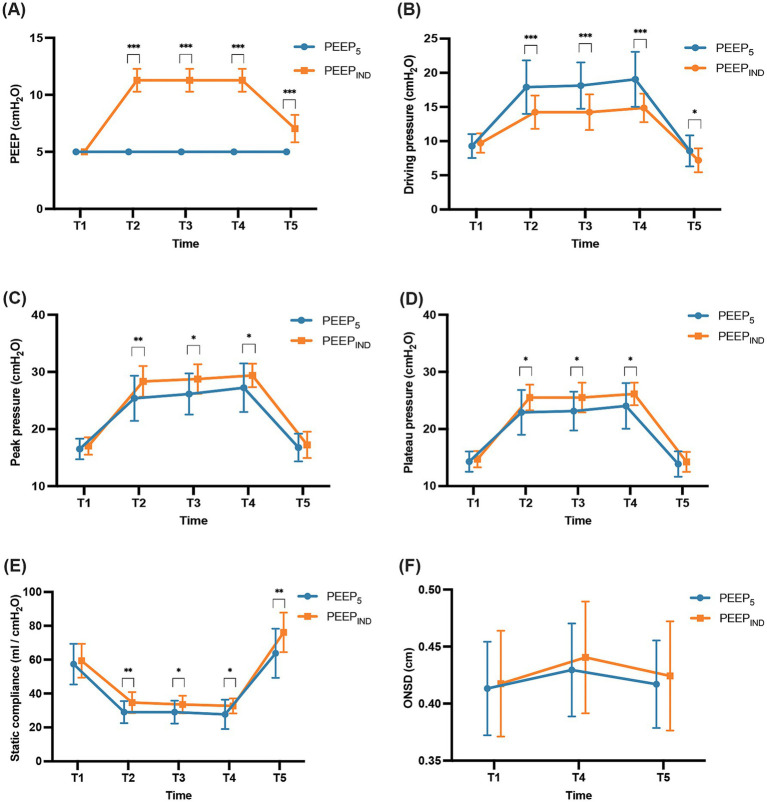
Intraoperative respiratory mechanics and ONSD. **(A)** PEEP, **(B)** driving pressure, **(C)** peak inspiratory pressure, **(D)** plateau pressure, **(E)** static compliance, **(F)** ONSD. T1, 10 min after intubation but before the first RM; T2, 10 min after pneumoperitoneum and Trendelenburg positioning; T3, 60 min after pneumoperitoneum and Trendelenburg positioning; T4, 120 min after pneumoperitoneum and Trendelenburg positioning; T5, at the end of the operation. ONSD, optic nerve sheath diameter. *p < 0.05, **p < 0.01, ***p < 0.001.

In comparison with the PEEP_5_ group, the PEEP_IND_ group showed significantly higher (*p* < 0.001) PEEP values during pneumoperitoneum in the Trendelenburg position (mean [standard deviation], 11.29 cmH_2_O [1.01 cmH_2_O]) and after deflation and repositioning to the supine position (mean [standard deviation], 7.05 cmH_2_O [1.20 cmH_2_O]), with mean differences (95% confidence interval [CI]) were 6.29 cmH_2_O (5.84–6.73) and 2.05 cm H_2_O (1.52–2.58), respectively. The results revealed a significantly lower driving pressure in the PEEP_IND_ group than that in the PEEP_5_ group. The mean differences in driving pressure were 3.67 cmH_2_O (95% CI, 1.63–5.71), 3.91 cmH_2_O (95% CI, 2.01–5.80), 4.19 cmH_2_O (95% CI, 2.20–6.19), and 1.39 cmH_2_O (95% CI, 0.12–2.65) at T2, T3, T4, and T5, respectively (PEEP_5_ vs. PEEP_IND_, *p =* 0.001, *p =* 0.000, *p =* 0.000, and *p =* 0.033, respectively) (Figure 4).

During pneumoperitoneum in the Trendelenburg position, peak airway pressure (*p =* 0.000) and plateau pressure (*p* = 0.000) were significantly higher in both groups, while lung compliance showed a reduction (*p* = 0.000). After deflation of the pneumoperitoneum and transition to the supine position, these parameters returned to baseline levels in the PEEP_5_ group (*p* > 0.05). In contrast, in the PEEP_IND_ group, peak airway and plateau pressures returned to baseline levels, while lung compliance showed an enhancement (*p =* 0.000). Compared with the PEEP_5_ group, the PEEP_IND_ group exhibited higher peak airway pressure (*p* = 0.025) and plateau pressure (*p* = 0.029) but superior lung compliance (*p* = 0.009) (Figure 4).

#### 3.3.2 ONSD

In this study, the results showed that the group*time interaction had no statistically significant effect on ONSD. Therefore, the main effects of the group and time factors on ONSD were analyzed separately. There was no significant difference in ONSD in both groups (*F* = 0.299, *p* = 0.591). The time factor had a statistically significant impact on ONSD (*F* = 36.329, *p* = 0.000). After pairwise comparison, a significant difference was observed between T1 and T4 (*p* = 0.000) with an MD of 0.020 (95% CI: 0.013–0.026) and between T4 and T5 (*p* = 0.000) with an MD of 0.014 (95% CI: 0.007–0.021). However, no significant differences were observed between T1 and T5 (*p* > 0.05) (Figure 4).

#### 3.3.3 Hemodynamics

Symptoms of hypotension were observed in almost all patients during the RM in supine position, even though the SVV% before the RM was less than 13. After completion of the RM, the blood pressure and cardiac index returned to normal. In contrast, no patient experienced hypotension when undergoing the RM in the Trendelenburg position. No statistically significant differences were detected in the hemodynamic measurements between the groups, which included mean arterial pressure, cardiac index, and stroke volume variation (*p* > 0.05) (Table 2). The two groups also showed no differences in the dosage of vasoactive drugs (*p* > 0.05) (Table 1).

**Table 2:** Comparisons of intraoperative hemodynamics between the two groups.

Variable	Group	T1	*P* value	T2	*P* value	T3	*P* value	T4	*P* value	T5	*P* value
MAP (mmHg)	PEEP_5_	81.19 ± 7.63	0.859	88.67 ± 10.49	0.881	80.52 ± 5.48	0.128	79.52 ± 7.01	0.322	83.19 ± 6.45	0.902
	PEEP_IND_	81.62 ± 7.91		88.19 ± 9.90		83.67 ± 7.47		81.52 ± 5.87		82.90 ± 8.39	
CI (L⋅min^−1^⋅m^−2^)	PEEP_5_	3.14 ± 0.24	0.401	2.87 ± 0.35	0.795	2.72 ± 0.19	0.271	2.63 ± 0.21	0.409	2.86 ± 0.25	0.536
	PEEP_IND_	3.07 ± 0.30		2.90 ± 0.41		2.82 ± 0.33		2.71 ± 0.41		2.91 ± 0.29	
SVV (%)	PEEP_5_	10.15 ± 2.00	0.594	9.29 ± 1.89	0.610	9.22 ± 2.92	0.413	9.95 ± 2.92	0.188	10.97 ± 3.10	0.512
	PEEP_IND_	10.52 ± 2.41		8.99 ± 2.05		8.59 ± 1.91		8.92 ± 1.97		10.39 ± 2.53	

Data are presented as mean ± standard deviation. MAP, mean arterial pressure; CI, cardiac index; SVV, stroke volume variation; T1, 10 min after intubation but before the first recruitment maneuver; T2, 10 min after pneumoperitoneum and Trendelenburg positioning; T3, 60 min after pneumoperitoneum and Trendelenburg positioning; T4, 120 min after pneumoperitoneum and Trendelenburg positioning; T5, at the end of the operation.

#### 3.3.4 Postoperative complication

In this study, four patients developed hypoxemia after extubation, two each in the PEEP_5_ and PEEP_IND_ groups, with no significant difference between the two groups (*p* > 0.05). In both groups, none of the patients required postoperative oxygen therapy on day 2 or later, received initial ventilatory support for a period longer than 24 h, required re-intubation and mechanical ventilation, or developed pneumonia or pneumothorax. Five patients developed POD, two in the PEEP_5_ group and three in the PEEP_IND_ group, with no significant difference between the two groups (PEEP_5_ vs. PEEP_IND_, n (%), 2[9.5] vs. 3[14.3], *p* > 0.05).

## 4 Discussion

The present study demonstrated that the implementation of individualized PEEP guided by driving pressure can yield enhanced intraoperative oxygenation in comparison with administration of fixed PEEP of 5 cmH_2_O. This finding is consistent with the conclusions of previous studies (4, 15, 25). RM can facilitate the reopening of atrophied alveoli, and PEEP has been shown to maintain this reopened state, thereby improving oxygenation. In the present study, a RM was performed after intubation in both groups to offset the pulmonary atelectasis that occurred during the intubation process. However, after pneumoperitoneum and Trendelenburg positioning, the oxygenation index was lower in the PEEP_5_ group than in the PEEP_IND_ group. This suggests that a fixed PEEP of 5 cmH_2_O was inadequate to counteract alveolar atrophy resulting from changes in position and intra-abdominal pressure and that individualization of PEEP enhances ventilation-perfusion matching, thereby improving oxygenation. Similarly, our results also failed to demonstrate a sustained beneficial effect on oxygenation after extubating, suggesting that the advantages of individualized PEEP may be limited to the intraoperative period. Girrbach et al. (4) found that in normal–weight patients, RM and PEEP_IND_ primarily improved intraoperative lung function, while atelectasis from mechanical ventilation, Trendelenburg positioning, and capnoperitoneum resolved after extubation in patients with PEEP of 5 cmH_2_O. This may further explain the loss of oxygenation advantage following extubation. Based on these findings, further investigation into the two groups undergoing periodic RMs is warranted, particularly their differential impacts on oxygenation and hemodynamics across different surgical positions.

The study employed volume-controlled ventilation, the most common mode in general anesthesia, which maintained consistent tidal volume despite fluctuations in intra-abdominal pressure and changes in patient positioning. In this study, peak and plateau airway pressures increased but compliance decreased in both groups during pneumoperitoneum in Trendelenburg positioning. In the PEEP_IND_ group, peak and plateau pressure levels were higher due to the PEEP_IND_ levels exceeding 5 cmH_2_O. Notably, the mean individualized PEEP in this study was lower than that observed in previous studies (4, 15), as we defined PEEP_IND_ as the minimal PEEP within the range corresponding to the lowest driving pressure. Despite this, driving pressure was lower in the PEEP_IND_ group than in the PEEP_5_ group, leading to improved pulmonary compliance. The reduction in driving pressure enhanced pulmonary mechanics, which may in turn have contributed to better oxygenation.

Cardiocirculatory depression during RM remains a primary anesthetic consideration, particularly in volume-depleted patients. In our study, despite performing RM after volume resuscitation, transient hypotension developed in most supine-positioned patients but resolved promptly post-maneuver. Importantly, no hypotensive episodes occurred during pneumoperitoneum in Trendelenburg position, attributable to augmented venous return. These findings emphasize the necessity of pre-RM volume optimization and vigilant hemodynamic monitoring. Furthermore, our study revealed similar hemodynamic profiles between PEEP_IND_ and PEEP_5_ groups, with comparable vasopressor needs, consistent with Girrbach et al. and Ma et al.’s findings (4, 5), demonstrating its safety for circulatory function. A multicenter study suggested that patients receiving a higher PEEP with the RM were more likely to experience hemodynamic instability (26), which was not consistent with the findings of our study. This discrepancy may be explained by the higher proportion of supine-positioned RM in high-PEEP patients in the previous study.

ICP elevation during pneumoperitoneum in the steep Trendelenburg position (16, 17, 21, 27) may result from increased intra-abdominal pressure and cephalad diaphragmatic displacement imparing cerebral venous drainage. Therefore, the application of individualized PEEP raises concerns about potential further ICP elevation. Studies reported that the application of 5 or even 8 cmH_2_O of PEEP did not increase the ONSD in comparison with that observed using zero PEEP during pneumoperitoneum in steep Trendelenburg positioning (28, 29). In the present study, both groups exhibited increased ONSD during pneumoperitoneum with steep Trendelenburg positioning. However, the implementation of PEEP_IND_, adapted to the surgical procedure, did not result in a further increase in ONSD when compared with PEEP_5_. In this study, the end-expiratory carbon dioxide concentration was maintained within the normal range as far as possible. As mentioned above, the two groups showed no notable differences in PaCO_2_, ensuring that the impact of carbon dioxide on cerebral blood flow was minimized to the greatest extent possible. This study found no significant differences in POD incidence between groups, suggesting that PEEP_IND_ may be safely performed in robot-assisted laparoscopic prostatectomy patients without preexisting intracranial hypertension. However, given the limited sample size, this conclusion should be interpreted cautiously, and larger multicenter studies are needed to validate these findings.

While multiple studies have established the correlation between driving pressure and PPCs (19, 20, 30), others have reported no significant reduction in PPCs with driving pressure optimization (15, 31). Aligning with the latter, our study found no significant PPCs reduction in PPCs within 7 days after surgery with driving pressure-guided PEEP, potentially due to the transient intraoperative oxygenation improvement. However, these null findings should be interpreted cautiously given our limited sample size, which may have underpowered the detection of clinically relevant differences in PPC rates.

Our study had some limitations. First, the sample size, calculated based on the primary outcome (PaO₂/FiO₂ ratio), may be underpowered to detect clinically meaningful differences in postoperative complications (POD and PPCs). Importantly, the systematic assessment of these secondary outcomes provides complementary data to our intraoperative monitoring parameters. These findings should be interpreted with caution and require confirmation in larger, multicenter trials. Second, in the present study, the minimal PEEP value within the specified range that corresponded to the lowest driving pressure was defined as the individualized PEEP. However, more research is required to determine whether the maximum PEEP value within the specified range will yield analogous results. Third, despite the fact that patients were enrolled in the study on the basis of a moderate or high risk of PPCs, as determined by their ARISCAT risk score, the actual risk score was lower than expected.

## 5 Conclusion

In conclusion, driving pressure-guided individualized PEEP improves intraoperative respiratory mechanics and oxygenation without compromising hemodynamic stability or increasing intracranial pressure in patients undergoing robot-assisted laparoscopic radical prostatectomy. While these findings demonstrate its intraoperative safety profile, the limited sample size precludes definitive conclusions regarding POD and PPCs. These null findings require cautious interpretation and warrant validation through larger multicenter trials.

## Data Availability

The original contributions presented in the study are included in the article/Supplementary material, further inquiries can be directed to the corresponding authors.
