# Supplementary figures and images for: Individualized positive end-expiratory pressure guided by driving pressure in robot-assisted laparoscopic radical prostatectomy: a prospective, randomized controlled clinical trial
Source: Front Med (Lausanne). 2025 Apr 22;12:1573150. doi: 10.3389/fmed.2025.1573150 (PMC12052569; doi:10.3389/fmed.2025.1573150)

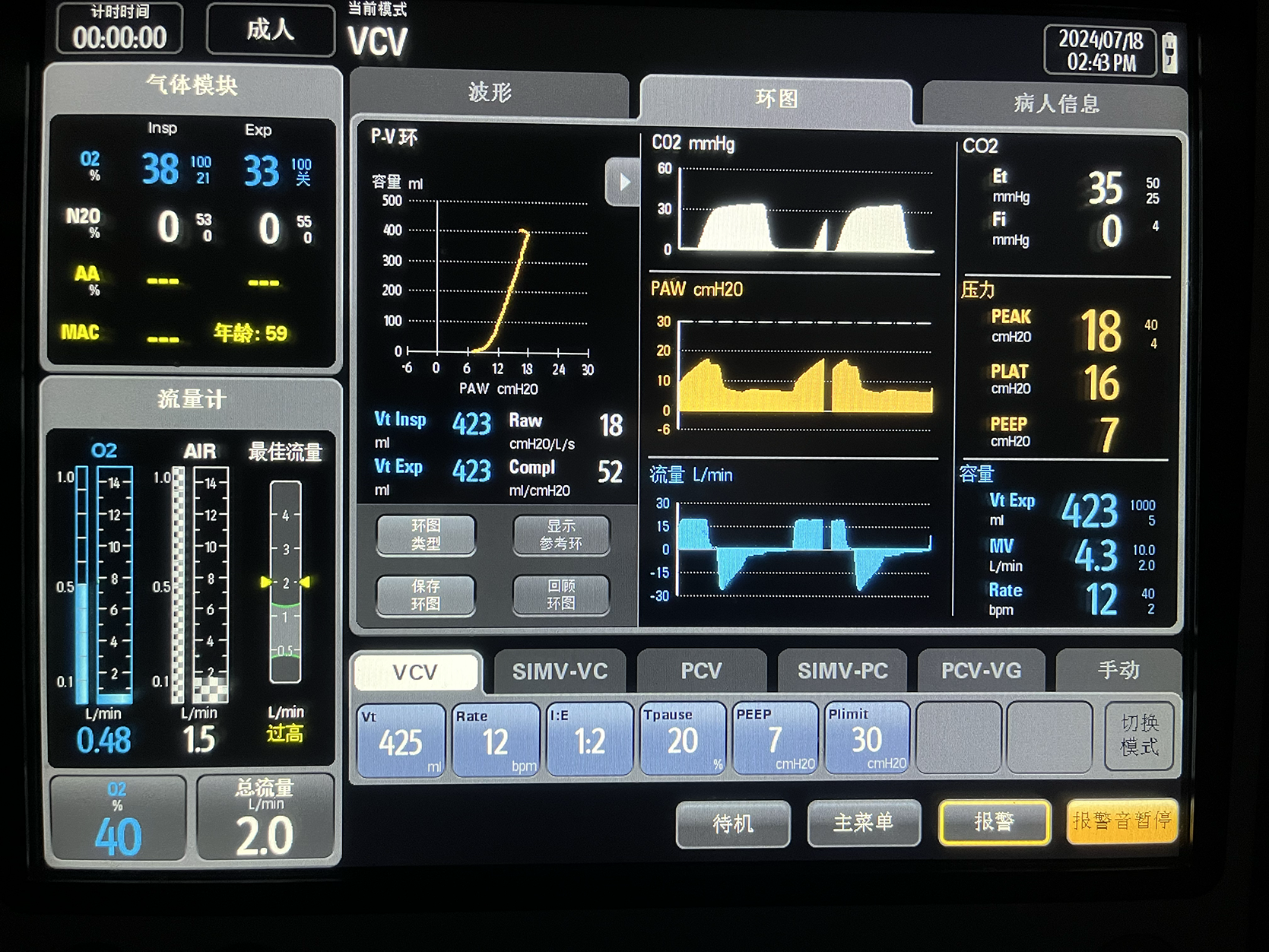

Supplement: Supplementary file 2 [file Presentation_1.zip › potograph/Supine position (1).tif]

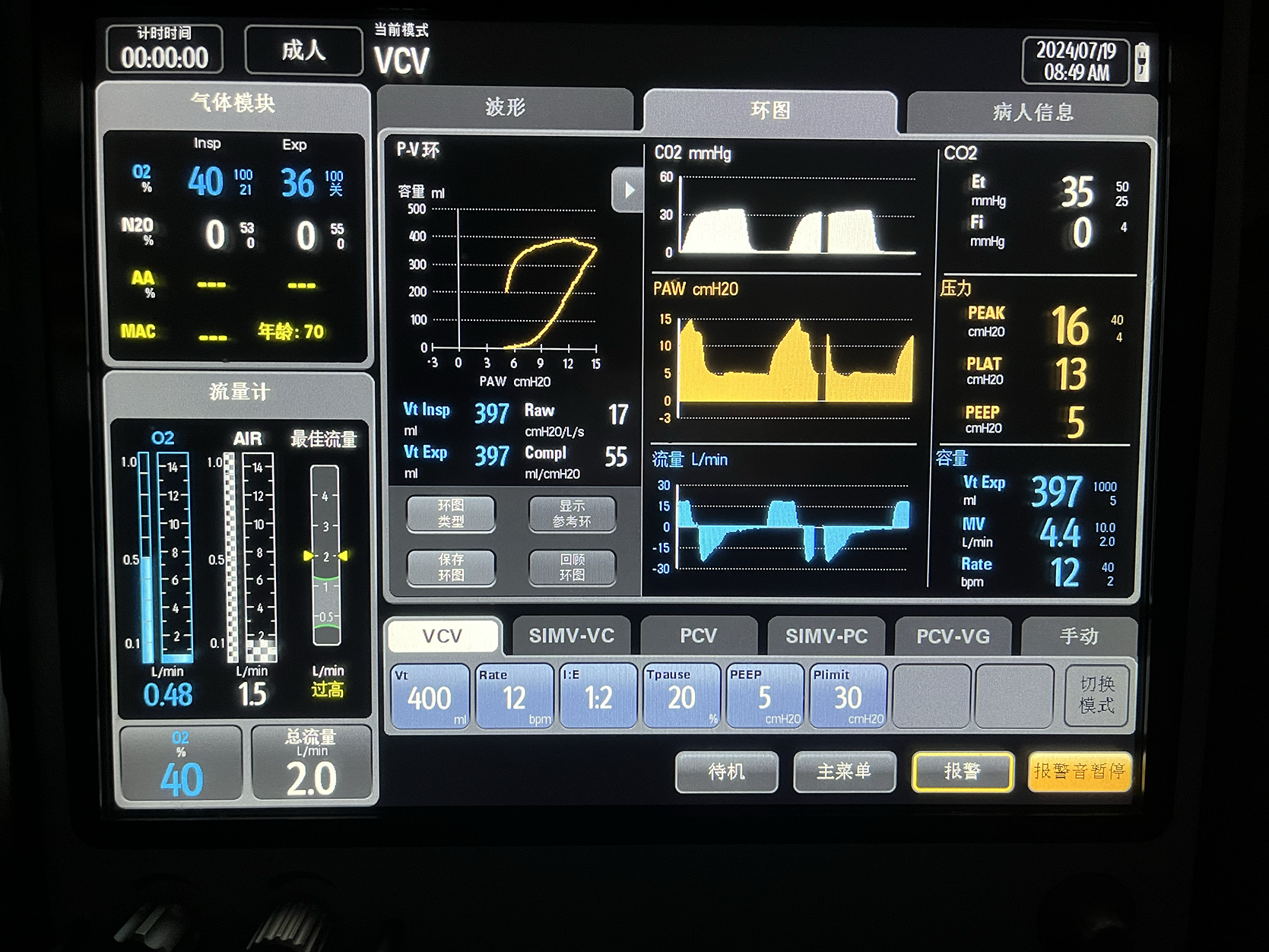

Supplement: Supplementary file 2 [file Presentation_1.zip › potograph/Supine position (2).tif]

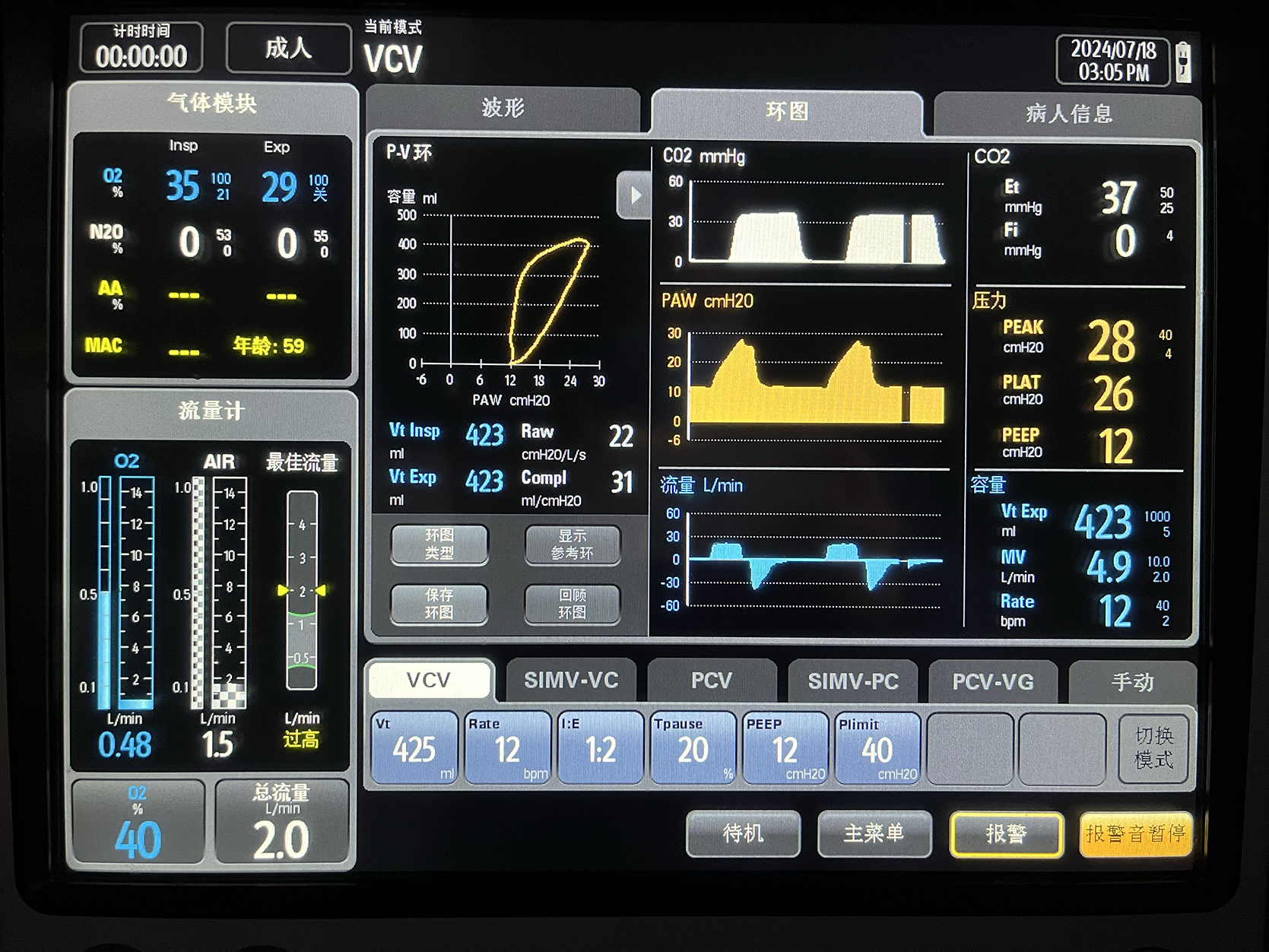

Supplement: Supplementary file 2 [file Presentation_1.zip › potograph/Trendelenburg position (1).tif]

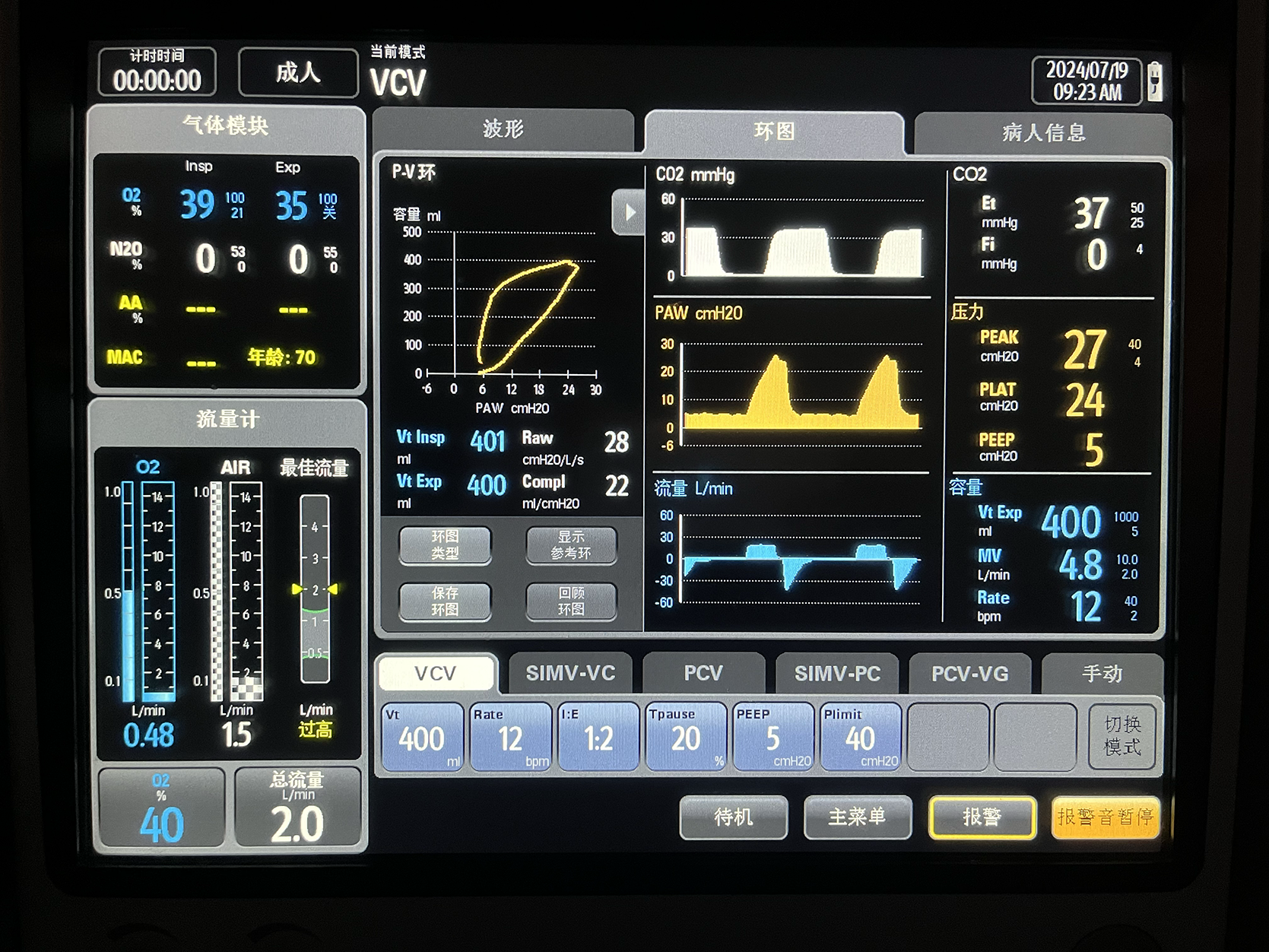

Supplement: Supplementary file 2 [file Presentation_1.zip › potograph/Trendelenburg position (2).tif]
