# Supplementary material for: Individualized positive end-expiratory pressure guided by driving pressure in robot-assisted laparoscopic radical prostatectomy: a prospective, randomized controlled clinical trial
Source: Front Med (Lausanne). 2025 Apr 22;12:1573150. doi: 10.3389/fmed.2025.1573150 (PMC12052569; doi:10.3389/fmed.2025.1573150)
Supplement: Supplementary file 3 [file Table_1.docx]

| Supplementary Table 1. PaO_2_/FiO_2_ ratios at five defined time points | | | | | |
| --- | --- | --- | --- | --- | --- |
| Group | T0 | T1 | T4 | T5 | T6 |
| PEEP_5_ | 379.52 ± 27.78 | 349.92 ± 33.53 | 329.50 ± 81.05 | 354.05 ± 90.47 | 344.63 ± 39.52 |
| PEEP_IND_ | 384.19 ± 40.17 | 354.52 ± 44.12 | 385.15 ± 70.95 | 413.45 ± 78.28 | 368.39 ± 42.78 |
| MD  (95% CI) | -4.664  (-26.21, 16.88) | -4.599  (-29.04, 19.84) | -55.649  (-103.16, -8.14) | -59.402  (-112.17, -6.64) | -23.765  (-49.45, 1.92) |
| *P* value | 0.664 | 0.706 | 0.023 | 0.028 | 0.069 |
| Data are presented as mean ± standard deviation. PEEP_5_, positive end-expiratory pressure of 5 cmH_2_O; PEEP_IND_, individualised positive end-expiratory pressure; T0, before intubation; T1, 10 min after intubation but before the first RM; T4, 120 min after pneumoperitoneum and Trendelenburg positioning; T5, at the end of the operation; T6, 30 min after extubation. MD (95% CI), mean differences (95% confidence interval) | | | | | |
